# ETView SL versus Macintosh Direct Laryngoscope for Endotracheal Intubation Amid Simulated COVID-19 Cardiac Arrest: A Randomized Crossover Study

**DOI:** 10.3390/jcm12155074

**Published:** 2023-08-02

**Authors:** Togay Evrin, Miroslaw Dabkowski, Michal Pruc, Jacek Hernik, Wojciech Wieczorek, Lukasz Chabowski, Pawel Wieczorek, Jaroslaw Chmielewski, Stepan Feduniw, Lukasz Szarpak

**Affiliations:** 1 Department of Emergency Medicine, Medical Faculty, Ufuk University, 06510 Ankara, Turkey; 2 Research Unit, Polish Society of Disaster Medicine, 05-806 Warsaw, Poland; 3 Department of Public Health, International Academy of Ecology and Medicine, 02000 Kyiv, Ukraine; 4 Institute of Outcomes Research, Maria Sklodowska-Curie Medical Academy, 00-136 Warsaw, Poland; 5 Department of Emergency Medicine, Medical University of Warsaw, 02-013 Warsaw, Poland; 6 Department of Public Health, Odessa International Medical University, 12042 Odessa, Ukraine; 7 Pediatric Intensive Care Unit (PICU), John Paul II Upper Silesian Health Centre in Katowice, 40-752 Katowice, Poland; 8 Institute of Environmental Protection—National Research Institute (IEP-NRI), 02-170 Warsaw, Poland; 9 Department of Public Health, International European University, 03187 Kyiv, Ukraine; 10 Department of Obstetrics, University Hospital Zurich, 8091 Zurich, Switzerland; 11 Department of Gynecology, University Hospital Zurich, 8091 Zurich, Switzerland; 12 Henry JN Taub Department of Emergency Medicine, Baylor College of Medicine, Houston, TX 77030, USA; 13 Research Unit, Maria Sklodowska-Curie Bialystok Oncology Center, 15-027 Bialystok, Poland

**Keywords:** endotracheal intubation, direct laryngoscopy, ETView SL, personal protective equipment, cardiopulmonary resuscitation, COVID-19

## Abstract

Airway management procedures, such as endotracheal intubation (ETI), pose a significant risk of aerosol generation, requiring robust personal protective equipment (PPE) against aerosol-generating procedures (AGP). This study aimed to assess the impact of PPE-AGP on intubation success rates, time to intubation, and glottic visualization using ETView and a standard Macintosh laryngoscope (MAC). A total of 52 physicians participated in this prospective, observational, randomized crossover study conducted in a medical simulation setting. Participants included COVID-19 patients with cardiac arrest scenarios with and without PPE-AGP who were intubated with ETView and MAC. During intubation without PPE-AGP, ETView showed a similar first-pass success rate (FPS) but had a shorter intubation time and better glottal hydration compared to MAC. In scenario B (with PPE-AGP), ETView outperformed MAC in FPS, initiation time, and glottic visualization. The use of PPE-AGP had little impact on ETView’s performance. However, it negatively affected the Macintosh laryngoscope, reducing FPS and glottic visibility. Participants found intubation with ETView easier in both scenarios. In conclusion, as compared to the Macintosh laryngoscope, ETView demonstrated higher performance under the circumstances of the simulation, especially when PPE-AGP was used.

## 1. Introduction

The COVID-19 pandemic brought attention to crucial difficulties facing patients suffering infectious illnesses and presented a challenge in numerous ways, including the safety of healthcare providers [1,2,3]. If a medical worker comes into close contact with an infected patient, then there is a possibility that they may also become sick. However, throughout the process of managing a patient’s airway, the danger of infection is significantly increased [4,5]. Some of the procedures for airway management, such as endotracheal intubation (ETI), bag-mask ventilation, non-invasive positive pressure ventilation, and surgical airways, have a considerable risk of creating aerosols [6]. Amidst the COVID-19 pandemic, infectious disease experts have highlighted the critical importance of bolstering personal protective measures, particularly when dealing with aerosol-generating processes. Healthcare professionals and frontline workers are more vulnerable during aerosol-generating processes, demanding a higher level of personal protective equipment (PPE) against aerosol-generating procedures (AGP). Higher-grade respirators, face shields, gowns, and improved ventilation systems are now required to protect the health and well-being of individuals on the frontlines and effectively restrict the virus’s spread. Such concentrated efforts to implement tight PPE-AGP standards can considerably contribute to lowering infection rates and, eventually, battling the COVID-19 pandemic [7]. During the early stages of the COVID-19 pandemic, the lack of readily accessible methods of personal protection was a major problem; however, this issue was resolved during the latter stages of the pandemic [8].

Endotracheal intubation is extremely important in the setting of cardiopulmonary resuscitation (CPR) [9]. When a patient goes into cardiac arrest while receiving CPR, efficient ventilation and oxygenation are critical for sustaining key organ function and boosting the odds of successful resuscitation. Airway management is essential for securing the patient’s airway, allowing oxygen to be delivered directly into the lungs, and providing regulated ventilation. Healthcare personnel can establish a patient’s airway, prevent aspiration, and provide accurate tidal volumes during artificial ventilation by placing an endotracheal tube through the trachea. Intubation also allows drugs to be administered and serves as a conduit for advanced airway management treatments such as capnography monitoring and defibrillation. As a result, in the context of cardiac resuscitation, prompt and successful intubation is critical for optimizing patient outcomes and enhancing overall resuscitation success.

During the course of the COVID-19 pandemic, guidelines for the treatment of airways have been published by a number of organizations and subject matter experts. These guidelines were developed with the intention of effectively treating COVID-19 patients while simultaneously decreasing the danger of viral transmission to medical workers [10,11,12]. When it comes to managing an individual’s airway, these recommendations continually emphasized how important it is to adopt suitable infection control measures, such as wearing appropriate personal protective equipment (PPE) against aerosol-generating procedures (AGP) while performing it [13].

**Figure 1:**
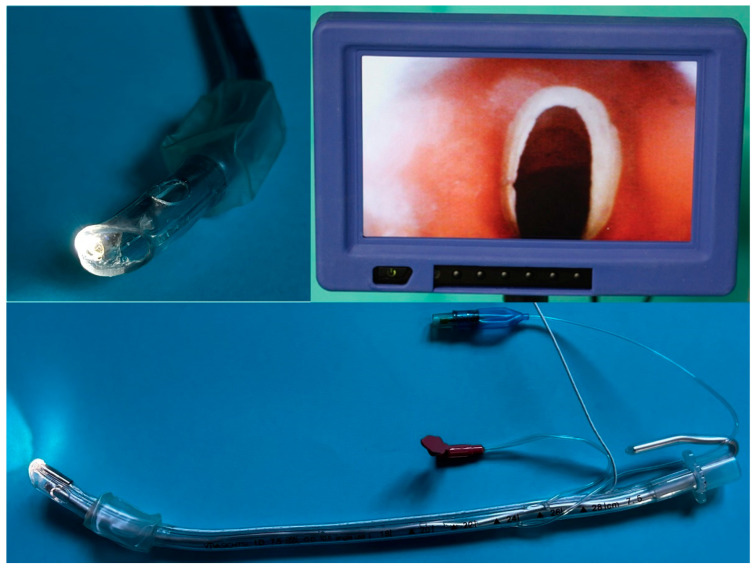
ETView SL laryngoscope.

ETView VivaSight, manufactured by ETView Ltd. in Misgav, Israel (ETView), is a recently developed endotracheal tube that is designed for a single use. The device enables continuous observation of the camera view by incorporating a high-resolution camera with a display monitor and a light source at the distal tip of the tracheal lumen (Figure 1).

Blood or any other fluids in the throat can pose a potential obstacle to the proper functioning of video laryngoscopes [14,15]. Nevertheless, the ETView overcomes this challenge with its innovative flushing system, enabling swift and effective in situ cleaning of the camera lens. This feature ensures uninterrupted and clear visualization during medical procedures, enhancing the device’s overall performance and safety [16,17]. It’s possible that the enhanced visualization capabilities may make it easier for less-experienced medical staff to effectively control patients’ airways in a variety of settings [18,19,20]. It remains unclear if the ETView is able to improve intubation conditions among physicians who are wearing personal protection equipment in general, resulting in an enhanced first-attempt success rate and a shorter intubation time in contrast to standard laryngoscopy.

During a simulated airway emergency, this research aimed to assess the first-pass success rate (FPS), time to intubation time, and glottic visualization using the Cormack–Lehane grade and percentage of glottis opening (POGO) score of physicians with and without personal protective equipment using ETView and a Macintosh laryngoscope.

## 2. Materials and Methods

The study was designed as a prospective, observational, randomized crossover study and was conducted in a medical simulation setting. The study protocol was approved by the International Review Board of the Polish Society of Disaster Medicine (no. 03.06.2022. IRB). The authors agreed to abide by the study protocol before the study began, and no changes were made to the study protocol during the study. The study included 52 physicians who participated in advanced cardiovascular life support courses taught by accredited instructors American Heart Association. Participants were recruited between July 2022 and May 2023, and all participants provided voluntary informed consent. All study participants had at least five years of experience in medicine. Physicians without a minimum of five years of professional experience or having a specialization in emergency medicine or anesthesiology were excluded from the study. Study participants who had any previous experience with or received training in the use of the ETView laryngoscope were also excluded from the study.

### 2.1. Scenario Design

Two laryngoscopes were evaluated in the study:

(a) ETVIEW SL (ETView Ltd., Misgav, Israel) as a representative of tubes with integrated cameras;
(b) Laryngoscope with a Macintosh blade no. 3 (MAC; HEINE Optotechnik GmbH & Co., KG, Gilching, Germany). Due to the high prevalence of this method, the Macintosh laryngoscope was chosen as the gold standard for intubation.

In both cases, intubation we use a CH 6 disposable intubation guidewire and a 7.5 mm internal diameter (ID) endotracheal tube. Each time, both the intubation tube and the stylet were moistened with a lubricant dedicated to simulator intubation.

Prior to the start of the study, all study participants took part in a 60 min standardized training course on methods of securing the airway in patients with infectious diseases. During the training, the correctness of intubation was discussed with each of the methods analyzed, and at the end of the training, the instructor demonstrated the correct intubation technique using ETView and MAC. The trainees then had a 10 min training session, during which they performed endotracheal intubation with the methods studied under normal airway conditions. For this purpose, the Laerdal^®^ Airway Management Trainer (Laerdal, Stavanger, Norway) was used. Training session was performed in normal airway scenario and performed without the PPE-AGP suit condition.

During the target trial, a SimMan^®^ 3G (Laerdal, Stavanger, Norway) advanced patient simulator was used to simulate an infectious disease patient (in this case, COVID-19) with cardiac arrest. The summator was placed on a flat floor in a room with a light intensity of 500 lx. To simulate the difficulties of cardiopulmonary resuscitation—the simulator’s chest was compressed using LUCAS3 in continuous mode [21].

**Figure 2:**
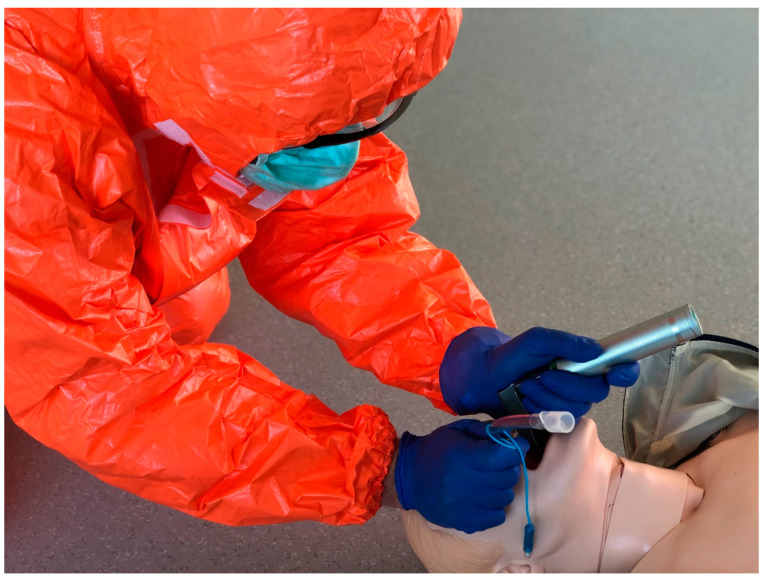
Endotracheal intubation in a PPE-AGP suit.

Participants performed endotracheal intubation with the laryngoscopes tested with and without PPE-AGP. To ensure the conditions for performing medical procedures with the PPE-AGP, the Tychem F chemical-resistant suit was employed, which protects against large concentrations of organic and inorganic chemical particles as well as those with a diameter of less than one micrometer (DuPont Personal Protection, Luxembourg). The subjects’ airways were protected with a protective mask equipped with an FFP2 filter (3M Aura Disposable Respirator, FFP2, Valved, 9312+, 3M Inc., Bracknell, UK), their eyes with protective googles and visors (MedaSEPT, Poznan, Poland), and their hands with double nitrile gloves (Figure 2).

**Figure 3:**
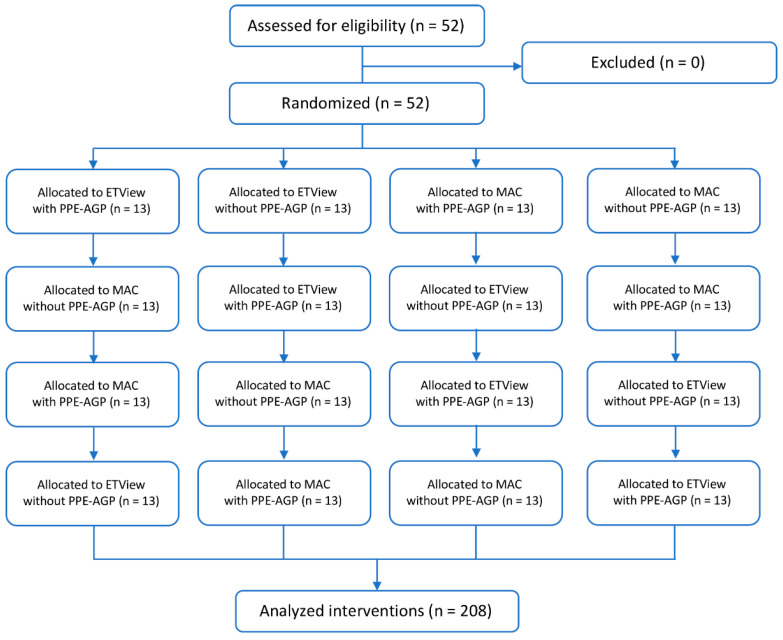
Randomization flow chart.

Both the order of participants and endotracheal intubation methods were randomized. For this purpose, the Research Randomizer program was used [22]. Study participants were divided into 4 groups: the first performed endotracheal intubation using ETView without PPE-AGP; the second using ETView with PPE-AGP condition; the third using MAC without PPE-AGP; and the fourth using MAC with PPE-AGP. After the intubation attempt, study participants had a 10 min break, after which they were intubated using a different method (Figure 3). Each participant in the study had a maximum of one intubation attempt by each method.

### 2.2. Outcomes

The key outcome was the first-pass success rate (FPS). FPS was measured when the ventilation indicators in the simulator confirmed the success of the ventilation endeavor. Secondary results include time to intubate, glottis visualization based on Cormack–Lehane classification [23], and percentage of glottis opening (POGO) score. The time to intubation was defined as starting when the laryngoscope blade was placed between the teeth and terminating until the first lung breathing occurred, as measured by the simulator’s sensors. The same investigator used a stopwatch each time. In addition, the ease of endotracheal intubation was assessed using a 10-point scale, where 1 meant easy intubation, and 10 meant difficult intubation.

### 2.3. Statistical Analysis

The sample size was calculated using G*Power 3.1 and a two-tailed *t*-test. A minimum of 39 physicians were necessary to achieve Cohen d = 0.8, alpha error = 0.05, and power = 0.95. We increased the minimum size of the study group to 52 people to provide a buffer in case of missing data or non-participation. The data were input into an Excel spreadsheet, and statistical analysis was performed using Statistica software version 13.4 EN (Tibco Inc., Tulsa, OK, USA). The data is shown as a mean (standard deviation (SD)) or as a number (with percentages provided), depending on the kind of data. Non-parametric tests, such as the Shapiro–Wilk and Lilliefors tests, were used in the absence of a normal distribution. A one-way ANOVA on rankings was performed to compare procedure times between groups, with a post hoc Bonferroni adjustment to account for multiple comparisons. In all relevant cases, two-sided statistical tests were run. A *p*-value of 0.05 was judged statistically significant in the research.

## 3. Results

Fifty-two physicians (37 men, 71.2%) with no clinical or simulation experience in intubation using ETView participated in the study. The average age of the participants was 42.1 years, and the average age of those working as a doctor was 16.9 years. Of the participants, 27 had specializations in internal medicine, 10 in family medicine, and 5 each in neurology, orthopedics and traumatology, and geriatrics, respectively. All participants in the study claimed clinical experience with intubation using direct laryngoscopy.

### 3.1. Intubation without PPE-AGP Scenario

**Figure 4:**
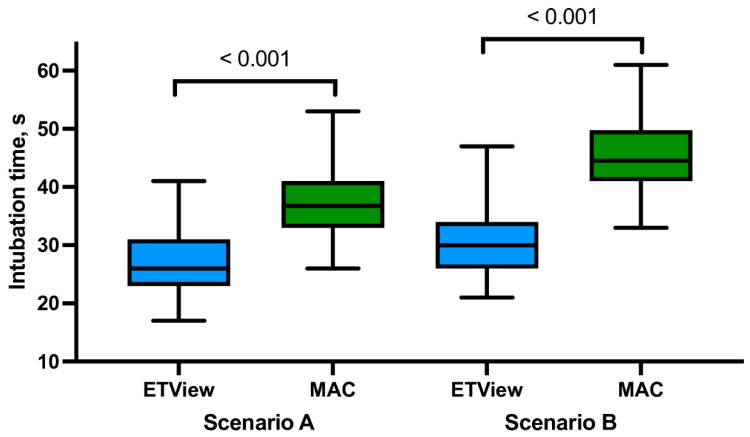
Intubation time using the intubation techniques under study.

During intubation in Scenario A (intubation without PPE-AGP), there were no statistically significant differences in FPS between intubation using ETView and MAC (92.4% vs. 82.7%, respectively; *p* = 0.187; Table 1). Intubation using ETView was associated, in comparison with MAC, with a shorter intubation time (27.1 ± 4.9 vs. 37.2 ± 5.7s; *p* < 0.001; Figure 4), better glottal hydration based on both Cormack–Lehane scales (*p* < 0.001) and a higher POGO score (*p* < 0.001). Study participants estimated that intubation using ETView compared to MAC was associated with statistically significantly easier intubation (4.1 ± 1.3 vs. 4.8 ± 1.4; *p* = 0.004).

### 3.2. Intubation with PPE-AGP Scenario

Intubation using ETView compared to MAC during scenario B (with PPE-AGP) was associated with significantly statistically better performance with respect to FPS (88.5% vs. 69.2%; *p* = 0.016; Table 2), initiation time (30.5 ± 5.3 vs. 45.2 ± 6.2; *p* < 0.001; Figure 3). Forty-six people intubating with ETView (88.5%) rated intubation with this method as 1–2 grade on the Cormack–Lehane scale, which was a statistically significantly better visualization of the glottis compared to intubation with MAC (21.2%; *p* < 0.001). Glottic visualization using ETView and MAC also showed a significant statistical advantage for ETView when assessed by POGO score (72.1 ± 15.3 vs. 38.3 ± 14.2, respectively; *p* < 0.001). The ease of intubation with ETView during intubation with the PPE-AGP scenario was rated by participants at 4.8 ± 1.5 points, compared to 7.1 ± 1.1 points during intubation with MAC (*p* < 0.001).

### 3.3. Impact of PPE-AGP on Intubation with ETView

The use of the PPE-AGP suit had no statistically significant effect on FPS during intubation using ETView (94.2% vs. 88.5%; *p* = 0.057). However, the use of the PPE-AGP suit, when compared with intubation without PPE-AGP, was associated with increased intubation time (30.5 ± 5.3 vs. 27.1 ± 4.9s, *p* = 0.001; Figure 3), while it did not significantly affect the degree of glottis visualization relative to the Cormack–Lehane scale (*p* = 0.079), as well as the POGO score (*p* = 0.535), or the ease of endotracheal intubation using ETView (*p* = 0.121).

### 3.4. Impact of PPE-AGP on Intubation with Macintosh Laryngoscope

Intubation with a Macintosh laryngoscope with and without PPE-AGP was associated with a statistically significant reduction in the success rate of the first intubation attempt (82.7% vs. 69.2%, respectively; *p* = 0.03). The use of PPE-AGP was also associated with a significant increase in the duration of endotracheal intubation (37.2 ± 5.7 vs. 45.2 ± 6.2s; *p* < 0.001) and a worsening of glottis visibility according to both the Cormack–Lehane scale (*p* < 0.001) and POGO score (*p* < 0.001). Study participants further demonstrated that intubation with PPE-AGP was easier to perform compared to intubation without PPE-AGP (4.8 ± 1.4 vs. 7.1 ± 1.1 pints, respectively; *p* < 0.001).

## 4. Discussion

The findings from our simulation randomized controlled trial affirm the influence of personal protective equipment (PPE) in aerosol-generating procedures (AGP) during airway management in the context of airway management in simulated scenarios carried out by physicians with a minimum of five years of relevant professional experience who are inexperienced in intubation. Excluded were specialists in the fields of emergency medicine and anesthesia, as well as individuals who have prior experience using the ETView laryngoscope or who have undergone training in its usage. Considering that physicians had no prior expertise with the topic of the research, the significance of our findings is further highlighted by this fact.

Our study highlights the advantages of the ETView laryngoscope over the Macintosh laryngoscope under the conditions of PPE-AGP use and under non-PPE-AGP use conditions by the first-pass success rate and secondary results that include time to intubate, glottis visualization based on Cormack–Lehane classification, and percentage of glottis opening (POGO) score.

The application of PPE-AGP has had a significant impact on the execution of numerous medical procedures [24,25], including airway management [26,27], intravascular insertions [28,29], and overall resuscitation quality [21,30]. These modifications have resulted in a range of effects, ranging from small annoyances to substantial performance hiccups. It is worth noting that airway management, which often requires endotracheal intubation or tracheostomy, is classified as a high-risk aerosol-generating procedure [31,32]. The use of PPE-AGP suits adds an extra layer of protection, safeguarding healthcare staff from infection [33]. However, these PPE-AGP outfits can present difficulties. For starters, they may restrict the operator’s field of vision, especially if visors or goggles become fogged, increasing the risk of procedural problems [34,35]. Second, they can induce pain or impede movement, potentially leading to lower manual dexterity and extended procedure durations [24,25]. Furthermore, medical workers may have heat strain symptoms when wearing PPE-AGP for extended periods of time or conducting medical operations that demand physical exertion, such as cardiopulmonary resuscitation [36,37].

In our study, both first-pass success rate (FPS) and time to intubation were found to be crucial metrics during emergent intubation, particularly in cases such as cardiac arrest, where every second counts [38,39,40]. ETView’s superior performance in terms of greater FPS and quicker time to intubation in both scenarios (with and without PPE-AGP) may be very useful in these emergency situations. Previous studies have shown that video laryngoscopy can enhance intubation success rates and shorten intubation times when compared to direct laryngoscopy [41,42]. Importantly, our findings show that using PPE-AGP had no statistically significant influence on FPS with ETView, implying that the limits imposed by PPE-AGP do not significantly reduce ETView’s effectiveness. This is an important consideration in the context of the present COVID-19 pandemic and any future infectious disease outbreaks, in which healthcare providers must weigh the need to protect themselves against the ability to perform key procedures effectively. There are various possible explanations for this. To begin, the ETView system, as a videolaryngoscope representation, is intended to address some of the constraints associated with direct laryngoscopy, such as restricted vision [20,43]. ETView’s visual aids can compensate for any potential vision impairment produced by the PPE-AGP suit, such as fogged goggles or face shields. It provides a direct, clear view of the patient’s airway without requiring the operator’s line of sight. Second, because the gadget is video-assisted, other team members can instruct and correct the operator’s actions in real time, even if the surroundings are noisy or visually impaired. This can help alleviate the communication challenges caused by PPE-AGP. Furthermore, the statistical insignificance may reflect the fact that medical professionals have altered their methods and developed their skills in response to the problems provided by PPE-AGP. During the epidemic, regular training sessions, simulation exercises, and learning from real-world experiences may have played a role in decreasing the potential impact on procedural success rates.

We discovered that using PPE-AGP increased the intubation duration with both ETView and MAC, implying that, despite the benefits of ETView, the protective equipment is still a hindrance to the operation. This emphasizes the significance of proper training and adaptation to perform procedures with PPE in order to avoid any potential delays. In terms of glottic visualization, we discovered that the Cormack–Lehane grade and POGO score were consistently higher when using the ETView laryngoscope over the Macintosh. This improved vision may have contributed to ETView’s greater performance in our trial, and it has been described in other studies as a benefit of video laryngoscopy. Interestingly, utilizing PPE-AGP had no effect on glottis visualization or ease of endotracheal intubation while using ETView, but it drastically impaired these metrics when using the Macintosh laryngoscope. This underscores ETView’s potential utility in circumstances where PPE-AGP is required. ETView’s increased visualization is due to its video-assisted nature. ETView displays real-time, clear, and magnified views of the patient’s airway on a screen, avoiding any potential visual obstruction from PPE-AGP, such as fogged goggles or face shields. This characteristic is especially useful during intubation, which necessitates precise placement of the endotracheal tube [44]. The MAC, on the other hand, is dependent on the operator’s direct line of sight. Any visual obstacle caused by the PPE-AGP could impair the operator’s view of the glottis, making intubation more difficult [45]. As a result, utilizing MAC in the setting of the PPE-AGP significantly worsens glottic visibility and ease of intubation [46,47]. These findings emphasize the need to adapt to the limits imposed by PPE-AGP and apply appropriate technologies to keep important operations successful. They also emphasize the potential value of video laryngoscopy equipment, such as ETView, in situations where PPE-AGP is required, providing healthcare personnel with a dependable technique of airway management without jeopardizing their personal safety. Furthermore, these findings may influence future airway management guidelines or protocols, particularly in cases involving viral disorders. The continued usage and study of video laryngoscopy devices may eventually lead to modifications in conventional practice, stressing their value in healthcare contexts requiring PPE use.

It is worth noting that while our study included people with at least five years of medical experience, they had no prior experience with ETView. This shows that with additional training and experience, the efficiency and success rates of ETView intubation may improve. To achieve a satisfactory view of the glottis and subsequent intubation, the MAC, a standard direct laryngoscope, requires the doctor to align the oral, pharyngeal, and laryngeal axes. Excellent hand dexterity, a good sense of spatial orientation, and a complete understanding of airway anatomy are required. The learning curve for MAC is usually steep at first because it requires a lot of practice to develop the requisite coordination and anatomical awareness. This learning curve gradually plateaus as physicians gain proficiency over time and with adequate hands-on experience. Videolaryngoscopes, on the other hand, provide a unique learning experience. By providing a direct and magnified view of the airway via a video screen, they obviate the need for precise alignment of anatomical structures. As a result, the intubation process is simplified, potentially making the learning curve less steep when compared to the MAC. Because of their intuitive design and immediate visual feedback, novices may gain skills with videolaryngoscopes more quickly [48,49,50,51,52]. It is crucial to note, however, that competence with videolaryngoscopes still necessitates a detailed understanding of airway anatomy as well as the ability to adjust the endotracheal tube based on the presented video image.

### Limitations

Our study also has certain limitations. Given that this study was conducted in a simulation setting, the results might not be directly translatable to real-world clinical scenarios. The realism of mannequin-based airway simulation has its limits, and conditions in actual patients, especially those with anatomical abnormalities or pathologies, may present greater challenges. However, only medical simulation allows such tests to be performed without risking harm to both the patient and the medical personnel who perform the endotracheal intubation. In addition, due to technical restrictions, we were unable to evaluate the dispersion of aerosols in our investigation. Thus, additional studies in clinical settings are needed to further validate these findings.

## 5. Conclusions

In conclusion, the ETView laryngoscope demonstrated superior performance in FPS, time to intubation, and glottic visualization under both conditions with and without PPE-AGP. It appears to be a valuable tool in the current pandemic and potential future scenarios requiring protective equipment, offering potential improvements in safety and efficiency for both physicians and patients. These findings, however, need to be further validated in real-world clinical settings.

## Figures and Tables

**Table 1:** Intubation characteristics in scenario without PPE-AGP.

Parameter	ETView	MAC	*p*-Value
FPS, n(%)	49 (94.2%)	43 (82.7%)	0.187
Time to intubation (s), mean (SD)	27.1 ± 4.9	37.2 ± 5.7	<0.001
Cormack–Lehane			
1	22 (42.3%)	2 (3.8%)	<0.001
2	26 (50.0%)	38 (73.2%)	
3	4 (7.7%)	10 (19.2%)	
4	0 (0.0%)	2 (3.8%)	
POGO score, mean (SD)	73.9 ± 16.0	60.2 ± 20.0	<0.001
Ease of intubation (1–10), mean (SD)	4.1 ± 1.3	4.8 ± 1.4	0.004

Legend: FPS: first pass success rate; MAC: Macintosh laryngoscope; POGO: percentage of glottis opening; SD: standard deviation.

**Table 2:** Intubation characteristics in a scenario with PPE-AGP.

Parameter	ETView	MAC	*p*-Value
FPS, n(%)	46 (88.5%)	36 (69.2%)	0.016
Time to intubation, mean (SD)	30.5 ± 5.3	45.2 ± 6.2	<0.001
Cormack–Lehane			
1	13 (25.0%)	0 (0.0%)	<0.001
2	33 (63.5%)	11 (21.2%)	
3	6 (11.5%)	34 (65.4%)	
4	0 (0.0%)	7 (13.4%)	
POGO score, mean (SD)	72.1 ± 15.3	38.3 ± 14.2	<0.001
Ease of intubation (1–10), mean (SD)	4.8 ± 1.5	7.1 ± 1.1	<0.001

Legend: FPS: first pass success rate; MAC: Macintosh laryngoscope; POGO: percentage of glottis opening; SD: standard deviation.

## Data Availability

The data that support the findings of this study are available on request from the corresponding author (L.S.).
